# Neonatal Oral Imitation in Patients with Severe Brain Damage

**DOI:** 10.1371/journal.pone.0003668

**Published:** 2008-11-07

**Authors:** Tohshin Go, Yukuo Konishi

**Affiliations:** 1 Department of Infants' Brain & Cognitive Development, Tokyo Women's Medical University, Shinjuku-ku, Tokyo, Japan; 2 Core Research for Evolutional Science and Technology (CREST), Japan Science and Technology Agency, Kawaguchi-shi, Saitama, Japan; James Cook University, Australia

## Abstract

**Background:**

Neonates reproduce facial movements in response to an adult model just after birth. This neonatal oral imitation usually disappears at about 2- to 3-months of age following the development of cortical control. There is controversy relating to the nature and neural basis of such neonatal imitation. To address this issue, we studied the relationship between oral imitation, primitive reflexes, and residual voluntary movement in patients with severe brain damage.

**Methods:**

Six male and six female patients with cerebral palsy, from 4 to 39 years, were included in this study. Oral imitation was examined when they were awake and looked at the experimenter. Patients were evaluated as performing oral imitation when they opened their mouth repeatedly without visual feedback regarding their own behavior in response to the experimenter's oral movement. Tongue or lip protrusion was not examined because none of patients were able to do those behaviors due to their physical disability. Rooting and sucking reflexes were also investigated as representatives of primitive reflexes.

**Results:**

Six patients (50%) performed oral imitation. Mouth opening was not observed repeatedly in response to other facial expression without opening the mouth such as surprise or smile, excluding the possibility of nonspecific oral reaction. They exhibited little voluntary movement of their extremities. Half of them also manifested at least one primitive reflex. No patients exhibiting residual voluntary movements of their extremities performed oral imitation or primitive reflexes.

**Conclusions:**

Oral imitation reappears in a similar way to primitive reflexes in patients showing severely impaired cortical function and little voluntary movement of their extremities due to severe brain damage, suggesting that neonatal oral imitation is mainly controlled by the subcortical brain region.

## Introduction

The mouth is an organ that humans can move actively just after birth, as observed in the sucking and rooting reflexes and spontaneous smile [1]. Oral movement such as sucking, opening of the mouth, and mouthing that constitutes mouth manipulation to investigate an object can already be observed even in the second trimester of human pregnancy by ultrasonography [2], [3]. Babies have been shown to reproduce lip protrusion, mouth opening, and tongue protrusion in response to an adult model within a few hours after birth [4], [5]. This is called neonatal (oral) imitation, being observed only in humans, great apes (chimpanzees) [6], and monkeys (rhesus macaques) [7]. Neonatal imitation usually disappears at about 2- to 3-months of age following the development of cortical control [8], [9]. Primitive reflexes also exist at birth or develop shortly after, and gradually disappear as the infant develops. In addition, primitive reflexes reappear in adults under certain conditions, like brain damage [10].

There is controversy relating to the nature and neural basis of such neonatal imitation. Some investigators suggest that human neonates can cross-modally process visual and motor information and detect the equivalent motor response [11]. An alternative explanation indicates that neonatal imitation is a specific and directly elicited response mediated by the same kind of mechanism based on primitive reflexes [12]. To address this issue, we performed the first investigation of the relationship between oral imitation, primitive reflexes, and residual voluntary movement in patients with severe brain damage to study whether oral imitation also reappeared in a similar way to primitive reflexes.

## Methods

Six male and six female patients with cerebral palsy, from 4 to 39 years, were included in this study. Seven patients had spastic quadriplegic, three had athetoid, and two had hypotonic types of cerebral palsy. All patients responded to auditory and visual stimulation with saccade or head turn to the stimulus. None of them were able to speak a single word. Intelligent scales were unmeasurable level in all patients. Their physical abilities are indicated in Table 1. None were ambulatory. Half of them exhibited almost no voluntary movement of their extremities. Some patients showed residual voluntary movement of their extremities such as reaching or holding an object for a short period. Oral feeding was possible with help in all as they were at least capable of swallowing, except for one case of tube feeding. The study was approved by the Ethics and Research committee of the hospital providing comprehensive care for the patients and informed written consent was obtained from the caregivers of them.

**Table 1:** Relationship between oral imitation, primitive reflexes, and residual voluntary movement in patients with cerebral palsy.

Age (years)	Gender	CP Type	Oral Imitation	Sucking Reflex	Rooting Reflex	Tendon Reflexes	Voluntary Movement
4	Female	Spastic Q	+	ND	ND	ND	Little
6*	Male	Spastic Q	+	+	−	3+	Little
9	Male	Athetoid	+	−	+	3+	Little
17	Male	Spastic Q	+	ND	ND	ND	Little
23	Male	Spastic Q	+	ND	ND	3+	Little
39	Female	Spastic Q	+	−	+	3+	Little
6	Male	Hypotonic	−	−	−	+	Reaching
11	Female	Hypotonic	−	−	−	3+	Reaching
20	Male	Spastic Q	−	−	−	ND	ND
32	Female	Athetoid	−	−	−	+	Holding
33	Female	Athetoid	−	−	−	+	Holding
34	Female	Spastic Q	−	−	−	3+	Reaching

**Figure 1:**
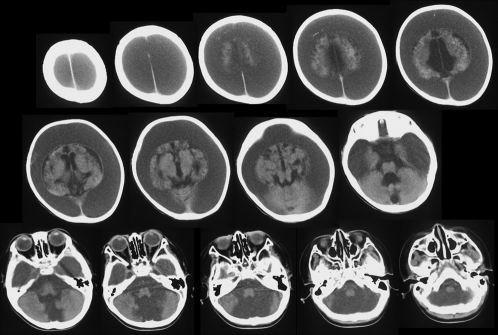
Brain computed tomography of a 6-year-old boy (indicated with * in Table 1). He suffered from severe cortical atrophy and massive subdural fluid collection.

**Figure 2:**
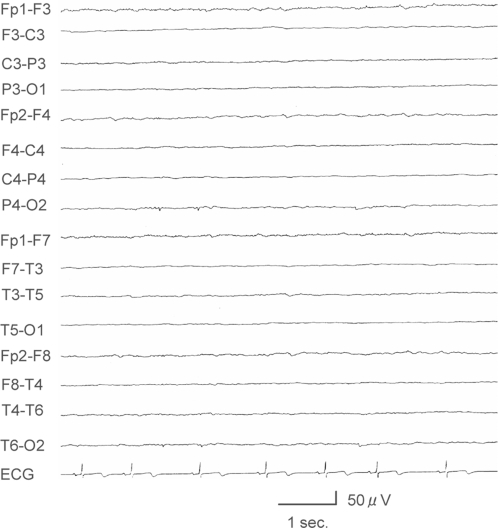
An awake electroencephalogram of a 6-year-old boy (indicated with * in Table 1). He had extremely low activity in all areas of cerebral cortex.

CP: Cerebral palsy, Q: Quadriplegia, ND: Not done, *: Brain computed tomography and an electroencephalogram of this patient are shown in Fig 1 and 2, respectively.

The experimenter sat face-to-face with each patient in the experimental room. The experimental procedure was similar to that of Meltzoff and Moore [4] with some modifications. Auditory stimulation such as a familiar song and the patient's name was used to sustain alertness and to encourage the patient to fixate visually on the experimenter's face. When the patient spontaneously looked at the experimenter, the experimenter widely and slowly opened the mouth three times during the 20-second test period. Patients were evaluated as performing oral imitation when they opened their mouth twice or more following the experimenter's mouth opening within the test period without visual feedback regarding their own behavior. Their response to other facial expression without opening the mouth such as surprise or smile was also examined to rule out the possibility that they nonspecifically open their mouth. Tongue or lip protrusion was not examined because none of patients were able to do those behaviors due to their severe physical disability. All examinations were performed for the first time in each patient when patients were awake with their eyes open and not excited. Therefore, learning or training could not influence the results. Rooting and sucking reflexes were also investigated as representatives of primitive reflexes because the mouth is their effector organ, similarly to oral imitation.

## Results

Six patients (50%), from 4 to 39 years, performed oral imitation (Table 1). Mouth opening was not observed repeatedly in response to other facial expression of the experimenter such as surprise or smile. They exhibited little voluntary movement of their extremities. Half of them also exhibited at least one primitive reflex. Brain computed tomography and an electroencephalogram of a representative patient are shown in Fig. 1 and 2, demonstrating severely reduced cortical structure and function. This patient is a 6-year-old boy. He was born at 39 weeks of gestation with a birth weight of 3350 g. When he was 2 months old, he showed frequent generalized convulsions and then coma due to bilateral subdural hemorrhage caused by child abuse. After this episode, he exhibited almost no voluntary movement of his extremities due to spastic quadriplegia. He showed oral imitation and the sucking reflex.

The other six patients, from 6 to 34 years, did not perform oral imitation. Most of them showed residual voluntary movement of their extremities such as reaching or holding an object for a short period. None of them exhibited any primitive reflexes.

## Discussion

The present study demonstrated for the first time that oral imitation reappeared in a similar way to primitive reflexes in patients showing severely impaired cortical function and little voluntary movement of their extremities due to severe brain damage. No patients exhibiting residual voluntary movements of their extremities performed oral imitation.

The neural basis for neonatal imitation is still unknown [13]. Therefore, the question of whether the oral imitation observed in this study is the same phenomenon as neonatal imitation can only be answered by a comparison of behavior observed both in newborns and patients with cerebral palsy. In neonatal imitation, the eliciting stimulus is visual circumoral (mouth and tongue) movement, and it induces the same action as a stimulus without touching the stimulus receiver. It resembles a primitive reflex. However, in primitive reflexes, the eliciting stimulus is mainly provided by touch, and it induces a different action from the stimulus. From this viewpoint, the oral imitation observed both in neonates and patients with cerebral palsy is considered the same phenomenon.

It has been proposed that neonatal imitation involves mainly subcortical regions including the superior colliculus via multimodal sensory mapping [14]. One of the bases for this hypothesis is that neonatal imitation disappears after 2 months of age following the development of cortical control. Automatic imitation behavior described in adults with frontal lobe damage [15] is considered another basis. In the present study, patients showing severely impaired cortical function and little voluntary movement of their extremities demonstrated a similar type of oral imitation to that observed in neonates, supporting this hypothesis. Further studies are necessary to clarify the mechanism of oral imitation in neonates and patients with severe brain damage.
